# Supplementary material for: Living with psychosis in West and Southeast Africa: SUCCEED Africa’s four-country situation analysis
Source: Glob Ment Health (Camb). 2025 Jan 8;11:e133. doi: 10.1017/gmh.2024.122 (PMC11729528; doi:10.1017/gmh.2024.122)
Supplement: Omobowale et al. supplementary material [file S2054425124001225sup001.docx]

**Appendix 1: Adapted SUCCEED Situation Analysis Template**

**BACKGROUND INFORMATION**

| **GENERAL INFORMATION** |  |
| --- | --- |
| Name of country |  |
| Name of district |  |
| Date situational analysis started |  |
| Date situational analysis complete |  |
| Completed by |  |
| Main sources of data^[[1]](#footnote-1)^ [refer to References for full list] |  |
| Notes on data collection process |  |

|  | **Description (*information on this section to be extracted from DHS and other similar national/regional reports*)** | **Baseline Situation** | | | **Data Source & Date** | **Notes** |
| --- | --- | --- | --- | --- | --- | --- |
|  |  | **National Level** | **Regional Level** | **District level** |  |  |
| **1.0** | **SOCIO-DEMOGRAPHIC INDICATORS** |  |  |  |  |  |
| 1.1 | Geography |  |  |  |  |  |
| 1.2 | Levels of governance |  |  |  |  |  |
| 1.3 | Population size |  |  |  |  |  |
| 1.4 | Population density |  |  |  |  |  |
| 1.5 | Proportion of persons who are:^[[2]](#footnote-2)^ | | | | | |
|  | 0-19 (children and adolescents) |  |  |  |  |  |
|  | 10-19 (adolescents) |  |  |  |  |  |
|  | 10-24 (young people) |  |  |  |  |  |
|  | 15-24 (youth) |  |  |  |  |  |
| 1.6 | Proportion living in rural areas |  |  |  |  |  |
| 1.7 | Proliferation of urban slums (*describe how much of a problem the proliferation of urban slums is* *in the area*) |  |  |  |  |  |
| 1.8 | Proportion of people in each major ethnic group within the country (%) |  |  |  |  |  |
| 1.9 | Language(s) |  | | | | |
|  | Official language(s) |  |  |  |  |  |
|  | Other major languages (%) |  |  |  |  |  |
| 1.10 | Major religions (%) |  |  |  |  |  |
| **2.0** | **ECONOMIC INDICATORS** |  |  |  |  |  |
| 2.1 | Adult Literacy rate |  | | | | |
|  | Female (%) |  |  |  |  |  |
|  | Male (%) |  |  |  |  |  |
| 2.2 | % of homes with improved water supply (including piped water, public taps, standpipes, tube wells, boreholes, protected dug wells and springs, rainwater, water delivered via a tanker truck or a cart with a small tank, and bottled water.) |  |  |  |  |  |
| 2.3 | % of homes with electricity supply |  |  |  |  |  |
| 2.4 | Major economic activity |  |  |  |  |  |
| **3.0** | **HEALTH INDICATORS** |  |  |  |  |  |
| 3.1 | Life expectancy |  |  |  |  |  |
|  | Female |  |  |  |  |  |
|  | Male |  |  |  |  |  |
| 3.2 | Infant mortality rate |  |  |  |  |  |
| 3.3 | Maternal mortality rate |  |  |  |  |  |
| **4.0** | **DISABILITY INDICATORS** |  |  |  |  |  |
| 4.1 | Proportion of persons with a physical disability |  |  |  |  |  |
| 4.2 | Proportion of persons with a mental disability |  |  |  |  |  |
| 4.3 | Prevalence of Schizophrenia |  |  |  |  |  |
| 4.4 | Prevalence of Bipolar Affective Disorder |  |  |  |  |  |
| 4.5 | Suicide rate (per 100,000) |  |  |  |  |  |
|  | Female |  |  |  |  |  |
|  | Male |  |  |  |  |  |
| 4.6 | Proportion of total YLD attributable to neuropsychiatric conditions |  |  |  |  |  |
| 4.7 | Proportion of total YLD attributable to schizophrenia |  |  |  |  |  |
| 4.8 | Proportion of the total YLD attributable to Bipolar Affective Disorder |  |  |  |  |  |
| 4.9 | (If available) Proportion of persons with psychosocial disability as reported by a study |  |  |  |  |  |

**SECTION 1: HEALTH (*to be completed by the national/regional/state focal person on mental health*)**

| **SN** | **Description** | **Levels of Hospital** | | | **Data Source & Date** | **Notes** |
| --- | --- | --- | --- | --- | --- | --- |
| **5.0** | **Pathways to Care and Support (Please describe)**  **Where do people typically first seek support for:** |  |  |  |  |  |
|  | **Physical health** |  |  |  |  |  |
|  | Mental healthcare (in the presence of psychiatric symptoms) |  |  |  |  |  |
|  | General Psycho-social support |  |  |  |  |  |
| **6.0** | **MENTAL HEALTH PROMOTION & PREVENTION**  ***(Answer “yes” or “no”)*** | ***Primary*** | ***Secondary*** | ***Tertiary*** |  |  |
| 6.1 | Are general health promoting services (e.g. information about engaging in physical activity and having healthy eating habits) available to persons with psychosocial disability? |  |  |  |  |  |
| 6.2 | Is access to general health check-ups (blood pressure, blood sugar etc.) readily available to persons with psychosocial disability? |  |  |  |  |  |
| 6.3 | Are the following mental health promotion and prevention programmes available? |  |  |  |  |  |
|  | Workplace mental health promotion programmes |  |  |  |  |  |
|  | School-based mental health promotion programmes such as school mental health clubs, mental health day, and school buddy |  |  |  |  |  |
|  | Parental/maternal mental health promotion |  |  |  |  |  |
|  | Early childhood stimulation programme |  |  |  |  |  |
|  | Mental health awareness/anti-stigma/human rights protection programmes |  |  |  |  |  |
|  | Violence prevention (especially for women and children) |  |  |  |  |  |
|  | Suicide prevention programmes |  |  |  |  |  |
| **7.0** | **MENTAL HEALTH CARE** | ***Primary*** | ***Secondary*** | ***Tertiary*** | Source |  |
|  | ***Treatment Coverage*** |  |  |  |  |  |
| 7.1 | Number of persons treated for psychosis per 100,000 within the last 12-month period |  |  |  |  |  |
| 7.2 | Average length of time from the onset of symptoms to the point of accessing mental health services (Duration of Untreated Psychosis) |  |  |  |  |  |
| 7.3 | Number of beds available for patients with mental illness compared with those with physical illness |  |  |  |  |  |
| 7.4 | Distance to the nearest general or specialist mental health service |  |  |  |  |  |
| 7.5 | Average duration of hospitalisation |  |  |  |  |  |
|  | ***Status of FACILITIES***  ***Answer “yes” or “no”*** |  |  |  |  |  |
|  | In comparison to physical health hospitals: |  |  |  |  |  |
| 7.6 | Is the building in a good state of repair (e.g. windows are not broken, paint is  not peeling from the walls)? |  |  |  |  |  |
| 7.7 | Does the building’s lighting (artificial and natural), heating and ventilation provide a comfortable living environment? |  |  |  |  |  |
| 7.8 | Are measures in place to protect people against injury through fire? |  |  |  |  |  |
| 7.9 | Do the sleeping quarters provide sufficient living space per service user and are not overcrowded? |  |  |  |  |  |
| 7.10 | Do men and women as well as children and older persons have separate sleeping quarters? |  |  |  |  |  |
| 7.11 | Does the sleeping quarters allow for the privacy of service users? |  |  |  |  |  |
| 7.12 | Are the bathing and toilet facilities clean and working properly? |  |  |  |  |  |
| 7.13 | Do the bathing and toilet facilities allow privacy? |  |  |  |  |  |
| 7.14 | Are there separate bathing and toilet facilities for men and women? |  |  |  |  |  |
| 7.15 | Are food and safe drinking-water available in sufficient quantities? |  |  |  |  |  |
| 7.16 | Are food and safe drinking-water of good quality? |  |  |  |  |  |
| 7.17 | Do food and safe drinking-water meet with the service users’ cultural preferences? |  |  |  |  |  |
| 7.18 | Are there ample furnishings, and are they comfortable and in good condition? |  |  |  |  |  |
| 7.19 | Is the layout of the facility conducive to interaction between and among service  users, staff and visitors? |  |  |  |  |  |
| 7.20 | Is service users’ privacy in communications respected? |  |  |  |  |  |
|  | ***Human Resources*** |  |  |  |  |  |
| 7.21 | Number of mental health professionals in the country |  |  |  |  |  |
| 7.22 | **Proportion working in the public sector:** |  |  |  |  |  |
|  | Psychiatrists |  |  |  |  |  |
|  | Clinical Psychologists |  |  |  |  |  |
|  | Psychiatric nurses |  |  |  |  |  |
|  | Psychiatric social workers |  |  |  |  |  |
|  | Clinical psychologists |  |  |  |  |  |
|  | Occupational therapists |  |  |  |  |  |
|  | ***Mental Health Politics, Policies & Plans*** |  |  |  |  |  |
|  | ***Political Support*** | ***National*** | | ***Regional*** |  |  |
| 7.23 | Political commitment for mental health services |  | |  |  |  |
| 7.24 | Is mental health specifically mentioned in general health policy? |  | |  |  |  |
|  | ***Mental Health Budget*** | ***National*** | ***Regional*** | ***District*** |  |  |
| 7.25 | What % of the total health budget is allocated to mental health? |  |  |  |  |  |
|  | ***Mental Health Policy/Plan*** | ***National*** | | ***Regional*** |  |  |
| 7.26 | **Existence of an officially approved mental health document within the last 5 years, such as:** |  | |  |  |  |
|  | Policy |  | |  |  |  |
|  | Plan |  | |  |  |  |
|  | Draft |  | |  |  |  |
| 7.27 | Any other officially approved document (older than 5 years)? |  | |  |  |  |
| 7.28 | **If present at all, what year was it last revised?** |  | |  |  |  |
|  | Policy |  | |  |  |  |
|  | Plan |  | |  |  |  |
|  | Draft |  | |  |  |  |
| 7.29 | **If present, describe how much of it has been implemented** |  | |  |  |  |
|  | Policy |  | |  |  |  |
|  | Plan |  | |  |  |  |
| 7.30 | **Does the policy/plan explicitly address issues of equity? Describe in relation to the following:** |  | | | |  |
|  | Gender |  | |  |  |  |
|  | Rural/urban residence |  | |  |  |  |
|  | Low socio-economic status |  | |  |  |  |
|  | **Does the policy/plan:** |  | |  |  |  |
| 7.31 | Promote transition toward community-based mental health services? |  | |  |  |  |
| 7.32 | Recognise that PWPD have the right to life on an equal basis with others? |  | |  |  |  |
| 7.33 | Allow for PWPD to be subjected without their free consent to medical or scientific experimentation? |  | |  |  |  |
| 7.34 | Recognise that persons with disabilities enjoy legal capacity on an equal basis with others in all aspects of life? |  | |  |  |  |
| 7.35 | Promote a full range of services and support to enable people live independently and be fully included in the community? |  | |  |  |  |
| 7.36 | Promote a recovery approach? |  | |  |  |  |
|  | Promote the participation of PWPD in decision-making? |  | |  |  |  |

| SN | **Description** | **National Level** | | **Regional Level** | | **Date Source & Date** | **Notes** |
| --- | --- | --- | --- | --- | --- | --- | --- |
|  | **Mental Health Law** |  | |  | |  |  |
| 7.37 | Is there existence of an officially approved mental health law? |  | |  | |  |  |
| 7.38 | If present, what year was it last revised? |  | |  | |  |  |
| 7.39 | If present, describe how much of it has been implemented? |  | |  | |  |  |
| 7.40 | Does the mental health law explicitly address issues of equity? (*Describe in relation to the following)*: |  | | | | |  |
|  | Gender |  | |  | |  |  |
|  | Rural/urban residence |  | |  | |  |  |
|  | Low socio-economic status |  | |  | |  |  |
| 7.41 | Does the mental health law: |  | | | | | |
| 7.42 | Promote transition toward community-based mental health services |  | |  | |  |  |
| 7.43 | Recognise that PWPD have the right to life on an equal basis with others? |  | |  | |  |  |
| 7.44 | Allow for PWPD to be subjected without their free consent to medical or scientific experimentation? |  | |  | |  |  |
| 7.45 | Recognise that persons with disabilities enjoy legal capacity on an equal basis with others in all aspects of life? |  | |  | |  |  |
| 7.46 | Promote a full range of services and support to enable people live independently and be fully included in the community |  | |  | |  |  |
| 7.47 | Promote a recovery approach |  | |  | |  |  |
| 7.48 | Promote the participation of PWPD in decision-making |  | |  | |  |  |
| 7.49 | Does the mental health law: |  | |  | |  |  |
| 7.50 | Stipulate that the building of the health facility must provide a welcoming, comfortable, stimulating environment conducive for active participation and interaction? |  | |  | |  |  |
|  | Stipulate that mental health services must be free from violence and abuse |  | |  | |  |  |
|  | Stipulate that mental health services must be free from exploitation |  | |  | |  |  |
|  | Promote transition towards community-based mental health services |  | |  | |  |  |
|  | Promote rights of PWPD to exercise legal capacity |  | |  | |  |  |
|  | Promote alternatives to coercive practices |  | |  | |  |  |
|  | Provide procedure for protection of the rights of PWPD and filing complaints to an independent body |  | |  | |  |  |
|  | Allow for regular inspection of human rights conditions in mental health facilities by an independent body |  | |  | |  |  |
| **8.0** | **REHABILITATION SERVICES** | ***Primary*** | ***Secondary*** | | ***Tertiary*** |  |  |
| 8.1 | Do persons with severe mental illness have access to rehabilitation services (such as vocational skills training programmes) ***(Answer “yes” or “no”) DESCRIBE*** |  |  | |  |  |  |
| 8.2 | What barriers to accessing rehabilitation services exist? |  |  | |  |  |  |

**SECTION 2: EDUCATION**

**(*Information from this section to be obtained from multiple sources including health records, PWPD, school heads, ministry of education*)**

**All responses to be in descriptive form unless studies available in country**

| **SN** | **Description** | **Secondary** | **Tertiary** | **Data Source & Date** | **Notes** |
| --- | --- | --- | --- | --- | --- |
| 9.0 | School drop-out rates amongst PWPD |  |  |  |  |
| 9.1 | School drop-out rates in the general population |  |  |  |  |
| 9.2 | Do students with psychosocial disability have equal opportunities for participation in school activities (e.g. Sports, drama etc.)? (*information from multiple sources: school heads, ministry of education, PWPD etc*) |  |  |  |  |
|  | **Rural** |  |  |  |  |
|  | Private |  |  |  |  |
|  | Public |  |  |  |  |
|  | **Urban** |  |  |  |  |
|  | Private |  |  |  |  |
|  | Public |  |  |  |  |
| 9.3 | Do students with psychosocial disability have equal opportunities for participation in decision making and leadership? (*information from multiple sources: school heads, ministry of education, pwpd etc.*) |  |  |  |  |
|  | **Rural** |  |  |  |  |
|  | Private |  |  |  |  |
|  | Public |  |  |  |  |
|  | **Urban** |  |  |  |  |
|  | Private |  |  |  |  |
|  | Public |  |  |  |  |
| 9.4 | Do students with psychosocial disability report experience of discrimination because of their disability? |  |  |  |  |
|  | **Rural** |  |  |  |  |
|  | Private |  |  |  |  |
|  | Public |  |  |  |  |
|  | **Urban** |  |  |  |  |
|  | Private |  |  |  |  |
|  | Public |  |  |  |  |
| 9.5 | Do schools have learning support such as flexible/individualised learning programmes for persons with psychosocial disability? |  |  |  |  |
|  | **Rural** |  |  |  |  |
|  | Private |  |  |  |  |
|  | Public |  |  |  |  |
|  | **Urban** |  |  |  |  |
|  | Private |  |  |  |  |
|  | Public |  |  |  |  |
| 9.6 | Are vocational training options available to persons with psychosocial disability? |  |  |  |  |
| 9.7 | Are professional training options available to persons with psychosocial disability? |  |  |  |  |
| 9.8 | Is access to continuing education available to persons with psychosocial disabilities? |  |  |  |  |

**SECTION 3: LIVELIHOOD**

**(*Information on this section to be obtained from sources which include PWPD and their families, published and unpublished reports*)**

**All responses to be in descriptive form**

| **SN** | **Description** | **Baseline Situation** | | **Date Source & Date** |
| --- | --- | --- | --- | --- |
|  |  | **Urban** | **Rural** |  |
| **10.0** | **SKILLS DEVELOPMENT**  ***(Answer “yes” or “no”)*** |  |  |  |
| 10.1 | Do persons with psychosocial disabilities (PWPD) have SAME access to acquiring digital/computer literacy skills AS PEERS in the general population? |  |  |  |
| 10.2 | Do PWPD feel they have access to EQUAL opportunities for acquiring or developing soft/life skills (e.g. communication skills, leadership skills etc.) COMPARED TO PEERS in the general population? |  |  |  |
| **11.0** | **EMPLOYMENT**  ***(Answer “yes” or “no”)*** |  | | |
| 11.1 | Do PWPD have access to funding opportunities to start up business COMPARED TO PEERS in the general population? |  |  |  |
| 11.2 | Is there a policy on employment that reserves a certain quota of an organisation’s workforce for PWPD? |  |  |  |
| 11.3 | Is there a policy/law that gives allowance to PWPD at work in form of reduced/proportionate workload, flexible work hours/conditions etc.? |  |  |  |
| 11.4 | Are PWPD paid a fair wage AS COMPARED TO PEERS WITH SIMILAR QUALIFICATIONS? |  |  |  |
| 11.5 | Is there a policy/law that protects the jobs of PWPD upon disclosure of mental health condition, relapse etc.? |  |  |  |
| **12.0** | **FINANCIAL SERVICES**  ***(Answer “yes” or “no”)*** |  | | |
| 12.1 | Do PWPD have access to financial services (loans, grants), livelihood/empowerment/poverty alleviation programmes COMPARED TO PEERS in the general population? |  |  |  |
| 12.2 | Are PWPD able to make decisions about how they spend their own money COMPARED TO PEERS in the general population? |  |  |  |
| 12.3 | Do PWPD know how to access financial services such as credit, insurance, grants, and savings programmes COMPARED TO PEERS in the general population? |  |  |  |
| **13.0** | **SOCIAL PROTECTION/WELFARE**  ***(Answer “yes” or “no”)*** |  | | |
| 13.1 | Are PWPD covered by social protection/welfare programmes? |  |  |  |
| 13.2 | If, available, do PWPD know how to access social protection measures? |  |  |  |
| 13.3 | Do PWPD access social welfare on an equal basis with peers with other disabilities? |  |  |  |
| 13.4 | Is there availability of supported housing (e.g. support for rent, special housing schemes) for PWPD? |  |  |  |

**SECTION 4: SOCIAL**

**(*Information on this section to be obtained from sources which include PWPD and their families, published and unpublished reports*)**

**All responses to be in descriptive form**

| **SN** | **Description** | **Baseline Situation** | | **Data Source & Date** |
| --- | --- | --- | --- | --- |
|  |  | **Urban** | **Rural** |  |
| **14.0** | **PERSONAL ASSISTANCE** |  |  |  |
| 14.1 | Do PWPD get to make their own decisions about the personal assistance (what assistance, who gives the assistance etc.) they need? (*please provide details*) |  |  |  |
| **15.0** | **RELATIONSHIP, FAMILY AND MARRIAGE** |  | | |
| 15.1 | Do PWPD get to make their own decisions about their personal & OTHER relationships? |  |  |  |
| **16.0** | **CULTURE AND ARTS** |  |  |  |
| 16.1 | Do PWPD get to participate in artistic, cultural or religious activities? AS COMPARED TO PEERS?? |  |  |  |
| **17.0** | **RECREATION, LEISURE AND SPORTS** |  |  |  |
| 17.1 | Do PWPD participate in mainstream recreational, leisure and sports activities? |  |  |  |
| 17.2 | Are there recreational, leisure and sports activities specifically for PWPD? |  |  |  |
| **18.0** | **RELIGIOUS INSTITUTIONS** |  |  |  |
| 18.1 | Do PWPD get any form of support from religious institutions? If yes, please describe |  |  |  |
| 18.2 | How much are PWPD allowed to participate in religious activities? Very much (b) Much (c) Little (d) Not at all (please explain) |  |  |  |
| **19.0** | **JUSTICE** |  | | |
| 19.1 | How much do PWPD know their legal rights e.g. right to vote, right to life, right to education, right to optimal healthcare, freedom of expression etc. AS COMPARED TO PEERS?   1. Very much (b) Much (c) Little (d) Not at all (please explain) |  |  |  |
| 19.2 | Do PWPD know how to access mechanisms of justice (e.g. law court, the police etc.) in cases of abuse or discrimination? |  |  |  |
| 19.3 | Does the law recognize severe mental illness as a form of disability? |  |  |  |
| 19.4 | Are there specific legal counsels available to help PWPD protect their rights and avoid stigmatization and discrimination at work or in the society? |  |  |  |
| 19.5 | Do PWPD feel valued as individuals by members of their community (Do they feel that other people respect them? Do they feel that others value you them as a person and listen to what they have to say? |  |  |  |
|  |  |  |  |  |

**SECTION 5: EMPOWERMENT**

**(*Information on this section to be obtained from sources which include PWPD and their families, published and unpublished reports*)**

**All responses to be in descriptive form**

| **SN** | **Description** | **Baseline Situation** | | | | **Data Source & Date** | |
| --- | --- | --- | --- | --- | --- | --- | --- |
|  |  | **Urban** | | **Rural** | |  |  |
| **20.0** | **ADVOCACY AND COMMUNICATION** |  | | | | | |
| 20.1 | Do PWPD think that the policies in the country provide people with disability equal rights as other people? |  | |  | |  | |
| 20.2 | Are PWPD organised effectively to communicate their wishes and objections to the authorities? |  | |  | |  | |
| 20.3 | Are PWPD well represented in advocacy groups and campaigns? |  | |  | |  | |
| **21.0** | **COMMUNITY MOBILIZATION** |  | | | | | |
| 21.1 | Do PWPD participate in awareness-raising or anti-stigma campaigns? |  | |  | |  | |
| **22.0** | **POLITICAL PARTICIPATION** |  | |  | |  | |
| 22.1 | Do PWPD engage in local or national politics and in civil society organizations compared to people without disability, or people with physical disabilities? |  | |  | |  | |
| 22.2 | Are PWPD or with a diagnosis of mental illness legally allowed to vote? |  | |  | |  | |
| 22.3 | Are PWPD welcomed as members of political or civil organizations? |  | |  | |  | |
| 22.4 | How do PWPD perceive their eligibility to vote? |  | |  | |  | |
| 22.5 | Are PWPD allowed to run for political offices? |  | |  | |  | |
| **23.0** | **SUPPORT GROUPS** |  | | | | | |
| 23.1 | Do peer support groups exist for PWPD? |  | |  | |  | |
| **24.0** | **DISABLED PEOPLE’S ORGANIZATIONS** | **National Level** | **Regional Level** | | **District Level** | | **Data Source & Date** |
| 24.1 | Are there recognised DPOs for people with psychosocial disabilities? |  |  | |  | |  |
| 24.2 | Do PWPD tend to identify in this way publicly and see value in belonging to a DPO? |  |  | |  | |  |
| 24.3 | Do PWPD feel that they are adequately represented by DPOs (e.g. Do PWPD feel their DPO representatives can help get justice in cases of abuse)? |  |  | |  | |  |
| 24.4 | Does the national disability federation include representatives of psychosocial disability DPOs? (please name national federation) |  |  | |  | |  |

**Appendix 2 : Distribution of 18 persons with professional and lived experience across the 4 countries.**

| **Country** | **Peer Researchers (Lived Experience)** | **Professionals** | **Background and experience** |
| --- | --- | --- | --- |
| Malawi | 1 | 3 | Social worker, Psychiatric Nurse |
| Nigeria | 1 | 4 | Psychiatrist, Community Physician, Psychiatry Resident , Research Associate |
| Sierra Leone | 2 | 3 | Psychologist, social workers |
| Zimbabwe | 2 | 2 | Psychiatrist, social workers |

1. Formative research may be conducted subsequently in order to obtain data on Items which are deemed highly important and relevant to the study, but for which data are not currently available [↑](#footnote-ref-1)
2. Country sites may amend to suit their focus and target population [↑](#footnote-ref-2)
